# Contextual factors associated with neonatal pain responses: clinical observational study

**DOI:** 10.3389/fped.2025.1508320

**Published:** 2025-03-20

**Authors:** Xinling Zhan, Nanxi Zhu, Bingjie Long, Zechuan Wang, Rui Miao, Gang Wang, Juan Chen, Chi Huang, Lu Xiong, Yi Huang, Simon Ching Lam, Lianhong Wang, Renli Deng

**Affiliations:** ^1^ Department of Nursing, Affiliated Hospital of Zunyi Medical University, Zunyi, Guizhou, China; ^2^ Department of Nursing, The Fifth Affiliated (Zhuhai) Hospital of Zunyi Medical University, Zhuhai, Guangdong, China; ^3^ Department of Nursing, Xiamen Children’s Hospital, Xiamen, Fujian, China; ^4^ Department of Basic Teaching, Zhuhai Campus of Zunyi Medical University, Zhuhai, Guangdong, China; ^5^ Zhuhai Zhongke Huizhi Technology Co., Ltd., Zhuhai, Guangdong, China; ^6^ Department of Neonatology, Affiliated Hospital of Zunyi Medical University, Zunyi, Guizhou, China; ^7^ School of Nursing, Tung Wah College, Hong Kong, Hong Kong SAR, China

**Keywords:** neonate, pain responses, pain assessment, contextual factors, multiple linear regression analysis

## Abstract

**Objectives:**

This study aimed to identify the contextual factors of neonatal pain responses and provide clinical medical staff with evidence regarding effective means of evaluating neonatal pain and strengthen clinical pain management.

**Methods:**

Two trained nurses independently used the Neonatal Infant Pain Scale (NIPS) to assess the pain scores of 198 neonates after they underwent painful medical procedures. Univariate linear regression analysis was performed to analyze the correlation between contextual factors and NIPS scores. Variables with statistically significant differences (*p* *<* 0.2) after univariate linear regression analysis were selected as independent variables, and the NIPS score was used as the dependent variable. Multiple linear regression was used to determine the salient factors associated with neonatal pain responses. This study was registered at the Chinese Clinical Trial Registry (ChiCTR2300074086).

**Results:**

Univariate linear regression analysis showed that the NIPS scores were associated with days after birth, types of painful procedures, Apgar scores at 1 min after birth, and gestational age (GA) (*p* < 0.2). Multiple regression analysis showed that Apgar score at 1 min after birth (*β* = 0.272, *p* < 0.001) and GA (*β* = 0.503, *p* < 0.001) were independent associated factors of neonatal pain responses. Neonates with low Apgar scores at 1 min after birth and younger GA had less pronounced pain responses.

**Conclusions:**

The Apgar score at 1 min after birth and GA affected the neonatal pain responses. In this regard, the current clinical method of pain assessment solely through observation of neonatal pain responses is occasionally inaccurate. The Apgar score at 1 min after birth and GA should be considered in determining the neonatal pain status and hence enhance the quality of neonatal pain management.

## 1 Introduction

The causes of neonatal pain include medical procedures, diseases, and trauma. Neonates who are hospitalized in the neonatal intensive care unit (NICU) receive more than a dozen painful treatments, such as endotracheal intubation, heel sticks, and venous and arterial punctures, every day on average (1–3). Neonatal diseases, such as intussusception, gastrointestinal perforation, and anal fissure, also produce pain symptoms. Repeated exposure to painful stimuli in neonates early in life leads to short- and long-term sequelae, including abnormalities in metabolism, brain development, and somatosensory and neural stress response systems (4, 5).

### 1.1 Neonatal pain assessment

Scholars have focused on developing an accurate evaluation of neonatal pain, which has been proven to be a challenge (6–8). At present, the pain status of neonates is evaluated through observation of their physiological and behavioral responses. Behavioral indicators commonly used to judge pain in neonates include facial expressions, physical activity, crying, and so on. The main physiological indicators used to judge pain comprise heart rate, respiration, oxygen saturation, blood pressure, and so on. However, these indicators differ in terms of the physiological and neurological development stages of neonates (9); as such, they do not fully reflect the pain status of neonates.

### 1.2 Influence of contextual factors on pain assessment

Contextual factors comprise the individual characteristics of neonates [e.g., sex, gestational age (GA), birth weight, etc.], length of hospital stay, mode of delivery, age after birth, therapeutic intervention, Apgar score, and so on. These factors can influence the pain responses of neonates (10–12). Therefore, the observation method for the responses of neonates for the assessment of their degree of pain may be biased (13, 14). Although the important role of contextual factors in pain assessment has been recognized, their influence is still often ignored in clinical work.

GA is often included in studies on the influence of pain responses on neonates. Numerous studies (10, 15–18) have shown a positive correlation between GA and pain reactions, and it was attributed to the progressive development of the neuromuscular system. Neonates with a high GA display complete development of the neuromuscular system (19, 20), as demonstrated through their physiology and behavior, such as facial expressions, body activity, heart rate, and so on, when experiencing pain (11, 21). A systematic review from Switzerland revealed that neonatal pain scores and behavioral responses increased with GA, which emphasizes the importance of considering GA in pain assessment (17). However, GA has no significant influence on pain responses (22, 23). Therefore, the correlation between GA and pain responses needs to be verified.

Gender is another important contextual factor in neonatal pain response. Although male infants show higher physiological pain responses (12), evidence confirming gender's effect on neonatal pain behaviors is insufficient (24). In addition, female neonates exhibit more facial expressions of pain, possibly due to differences in pain processing or expression between genders (25). Conversely, other studies found no significant correlation between gender and pain responses (26–28).

The relationship between the Apgar score and pain responses has also yielded inconsistent results. One study suggested the positive correlation of the Apgar score at 1 min after birth with the facial score (29), and another study implied its negative correlation with the pain score (12). Furthermore, Apgar scores at 5 min after birth have been positively associated with behavioral responses (12), including frowning and deepening of the nasolabial groove, but not with eye squeezing, heart rate, and oxygen saturation (30). Another research showed that infants with lower Apgar scores displayed more motor stress cues but less facial activity after painful procedures (29).

Moreover, some studies have incorporated contextual factors, such as the mode of delivery (31–33), birth weight (27, 33), and postnatal age (34). However, the findings remain inconsistent. One research found that a high birth weight considerably reduced pain responses during immunization (33), and another reported no significant correlation between birth weight and pain responses (29). In addition, endotracheal intubation, femoral vein puncture, and adhesive removal were identified as the most painful procedures (35), and higher pain scores were reported for venipuncture, heel sticks, and intramuscular injection (36).

In conclusion, a few studies have been conducted on other contextual factors that influence neonatal pain responses, and the results were inconsistent (17). Although various factors were identified, a discrepancy was observed in the findings related to insufficient sample size and the on-site assessment of environmental effects. This study aimed to identify the contextual factors of neonatal pain responses and provide clinical medical staff with evidence regarding effective means of evaluating neonatal pain and strengthen clinical pain management.

## 2 Methods

This cross-sectional study complied with the Strengthening the Reporting of Observational Studies in Epidemiology (https://www.equator-network.org/). In this work, a camera was used to record the pain responses of neonates undergoing painful medical procedures. After viewing the recorded videos, trained nurses scored the neonates' pain using the Neonatal Infant Pain Scale (NIPS). The data on neonatal contextual factors were collected, and the correlation between the contextual factors and pain scores was analyzed to determine the contextual factors affecting neonatal pain responses.

### 2.1 Participants

A total of 209 neonates admitted to the NICU at the Affiliated Hospital of Zunyi Medical University from October 2022 to December 2022 were selected. The inclusion criteria consisted of the following: (a) patients ≤28 days old; (b) patients who underwent painful procedures for diagnosis or treatment; (c) patients with parents or legal guardians who were informed of the study and agreed to participate. The exclusion criteria included the following: (a) patients in a coma; (b) patients in sedation and analgesia; (c) patients with abnormal craniofacial deformity; (d) patients whose face was covered for treatment, such as wearing a phototherapy eye mask, a helmet for nasal oxygen tube fixation, and tracheal intubation; (e) patients who were critically ill and needed immediate rescue.

### 2.2 Measures

#### 2.2.1 Video recording

The video recordings for this investigation were performed in the NICU of the Affiliated Hospital of Zunyi Medical University, a class-A tertiary facility with 2,860 patient beds and 4,352 staff. The hospitals with that grade are the top tier in the Chinese hospital classification system, known for their comprehensive healthcare services, advanced medical equipment, and high-quality medical staff. The current study venue served as the primary critical care facility for neonates in Guizhou Province and Zunyi City. The neonatal department comprises 120 beds and serves more than 3,000 patients each year.

When neonates undergo painful procedures, the environment is noisy, and the assessor cannot fully focus on observing neonatal pain, which result in bias in pain assessment. Video recording was used in the present study, which allowed the assessor to repeatedly view the videos and carefully observe the pain status of the neonates prior to making a final judgment.

The following steps were required before video recording. First, data collection was carried out every morning after the nurses completed their nursing practices, such as feeding, changing diapers, and morning care, and placed the neonates in a comfortable state to ensure the absence of interference from other uncomfortable events. Second, prior to conducting painful medical procedures, the incubator was adjusted to appropriate temperature and humidity, and the neonates' whole body was exposed. Third, the neonates' heads were adjusted and fixed to ensure that the face, body, and limbs can be photographed from the front. The neonates were in a calm state before their operation. Finally, the nurses ensured that the blood oxygen saturation probe was fixed and that the heart rate and blood oxygen saturation were normal.

A hand-held video camera was used to film the whole body of the neonates at a top-down angle and record the neonates’ responses to pain. Recording was conducted from 2 min before to 2 min after the painful medical procedure.

#### 2.2.2 Data collection involving contextual factors

Our research team previously established a neonatal pain-response variable set, which was used in literature review, panel meeting, and Delphi studies to reach consensus on contextual factors that may affect neonatal pain response (37). Therefore, based on this variable set, this study collected the contextual factor data of neonates, including their gender, GA, birth weight, length of hospital stay, delivery mode, postnatal age, type of painful procedure, and Apgar scores at 1 min after birth. The Apgar scores at 5 min after birth were excluded from the analysis due to the ceiling effect (approaching a mean score of 10) and their alteration with suitable clinical interventions (in cases of low Apgar scores at 1 min).

#### 2.2.3 Pain assessment

##### 2.2.3.1 Pain response assessment tool

In this study, the NIPS was used to assess the neonatal pain responses. This scale was developed by Professor Lawrence's team in Canada for the evaluation of acute irritant pain in neonates (38). The NIPS comprehensively assesses the pain status of neonates in terms of facial expressions, crying, breathing patterns, upper and lower limb movements, and wakefulness. Except for the crying index, which is divided into three grades (0–3 points), the other indicators comprised two grades (0–2 points). The total pain score is determined by summing all the scores. Scores of 0–2, 3–4, and 5–7 indicate little or no pain, moderate pain, and severe pain, respectively. The scale has been tested in neonatal populations in China, Brazil, Thailand, and other countries (39–41). It shows good reliability and validity and is easy to understand and use. In neonatal pain management guidelines and expert consensus developed by neonatal associations in the United States, Canada, and China, the NIPS is listed as a recommended assessment tool (5, 42, 43). Thus, in the present study, this scale was used as the gold standard for the assessment of the overall neonatal pain responses.

##### 2.2.3.2 Assessor training

Training on the use of the NIPS was carried out through previous clinical observations of neonatal pain responses, an interpretation of related guidelines for neonatal pain management, and evaluation tools for neonatal pain. Two nurses from the NICU were trained, and after training, they independently evaluated 20 randomly selected pain videos of neonates on the spot. After one week, these evaluators conducted another independent evaluation. Inter- and intragroup assessment consistencies were calculated to achieve a satisfactory agreement before data collection.

##### 2.2.3.3 Assessment methods

After daily video recording, the two nurses repeatedly watched the pain videos and independently assessed the pain of each neonate using the NIPS scale. The data they obtained were compared. In the case of any disagreement, the nurses returned to the original videos to discuss and decide the pain score together.

##### 2.2.3.4 Evaluation of consistency

Several nursing assessors performed the participants' pain assessments in clinical settings. For consistency, intergroup consistency, which refers to the agreement on the pain assessment of a participant between two assessors, and intragroup consistency, which refers to the agreement on the pain assessment of the same neonate twice by an assessor one week apart, was used prior to data collection. The intraclass correlation coefficient (ICC) was used to compute the coefficients of inter- and intragroup assessment consistency of the assessors. The ICC values indicate the consistency of the results: ICC > 0.75 indicates a high consistency; ICC = 0.4–0.75 denotes a good consistency; ICC < 0.4 implies a poor consistency.

#### 2.3.2 Statistical analysis

SPSS 26.0 was used in data analysis. The measurement data were represented by mean with standard deviation (SD) for normal distribution and by median [Q1–Q3] for nonnormally distributed variables. Count data were expressed as frequency (n) and percentage (%).

Univariate linear regression was performed to analyze the relationship between contextual factors and neonatal pain scores. Conservatively, multiple linear regression analysis was conducted on variables with statistically significant differences (*p* < 0.2) (44), with *p* < 0.05 as the standard significant value used to indicate independent influencing factors of neonatal pain responses.

### 2.4 Ethical approval

The ethical review received approval from the Biomedical Research Ethics Committee of the Affiliated Hospital of Zunyi Medical University (KLLY-2021-048). The registration was completed at the Chinese Clinical Trial Registry (ChiCTR2300074086). The content and purpose of the study were explained to the guardians of all participants, who provided informed consent.

## 3 Results

### 3.1 Consistency test

The two trained assessors used the NIPS to conduct independent evaluation of 20 randomly selected pain videos. The ICC of intergroup consistency was 0.889. Using the NIPS, assessors 1 and 2 independently rated the 20 videos again after one week. The ICCs of the intragroup consistency reached 0.903 and 0.898 (Table 1). All the ICC values were higher than 0.75, which implies satisfactory inter- and intragroup consistencies.

**Table 1:** Consistency of NIPS scores between the two evaluators.

Item	ICC correlation coefficient	95% confidence interval	
		Lower limit	Upper limit
Consistency between two evaluators	0.889	0.719	0.959
Intragroup consistency of evaluator 1 (after 1 week)	0.903	0.756	0.962
Intragroup consistency of evaluator 2 (after 1 week)	0.898	0.743	0.960

### 3.2 Demographics and associated factors

#### 3.2.1 Demographic information

A total of 209 neonatal pain videos were collected. Eleven poor-quality videos (e.g., facial reflection, body occlusion, blood oxygen probe falling off, etc.) were removed because of their possible effect on the accuracy of the results. A total of 198 videos were retained for analysis.

The 198 neonates had a mean GA of 254 (range of 237–272) days. The neonates included 100 males (50.5%) and 98 females (49.5%). A total of 54 neonates (27.3%) were delivered vaginally and 144 (72.7%) through cesarean section. The median length of hospital stay was 6 (range = 4–11) days. The median age after birth was 7.5 (range = 4–12.25) days. About 80.3% of the neonates suffered from complications. Blood was collected from heel sticks and fingertips of 77 (38.9%) and 76 (38.4%) neonates, respectively. These blood collection procedures were the most common types of painful medical procedures in this study. Blood was drawn from the brachial artery and femoral vein in 6 (3.0%) and 3 (1.5%) neonates, respectively. Among the remaining neonates, 11 (5.6%) received intravenous indwelling needle punctures, and 25 (12.6%) received radial artery draws. Table 2 shows the general information on the neonates.

**Table 2:** Demographic information (*N* = 198).

Characteristic	Specific content	Mean ± SD/median [Q1– Q3]	Number (*n*)	Percent (%)
Sex	Male		100	50.50
Length of stay		6 [4–11]		
Delivery mode	Vaginal delivery		54	27.30
GA (days)		254 [237–272]		
Postnatal age		7.5 [4–12.25]		
Birth weight (kilogram)		2.51 ± 0.81		
Apgar score at 1 min after birth		10 [9–10]		
Type of painful procedures	Heel stick		77	38.90
	Fingertip blood collection		76	38.40
	Intravenous indwelling needle puncture		11	5.60
	Radial artery draw		25	12.60
	Brachial artery draw		6	3.00
	Femoral vein draw		3	1.50
Complications	Yes		159	80.30
Disease diagnosis	Neonatal respiratory distress syndrome		40	11.70
	Neonatal pneumonia		128	37.50
	Hypoxic–ischemic myocardial damage		3	0.90
	Premature infant		105	30.80
	Neonatal hyperbilirubinemia		14	4.10
	Neonatal ABO hemolytic jaundice		4	1.20
	Neonatal hypoglycemia		31	9.10
	Neonatal necrotizing enterocolitis		1	0.30
	Metabolic acidosis		2	0.60
	Neonatal anemia		3	0.90
	Neonatal septicemia		4	1.20
	Neonatal thrombocytopenic purpura		6	1.70

#### 3.2.2 Univariate and multiple linear regression analysis

Univariate linear regression analysis was performed to determine the association among the eight contextual factors and pain scores. Days after birth, types of painful operation, Apgar score at 1 min after birth, and GA were associated with the neonatal pain scores at a significant value of 0.2. The results are shown in Table 3.

**Table 3:** Univariate linear regression analysis of contextual factors and neonatal pain scores.

Predictors	Unstandardized coefficient		Standardized coefficient	*t*	*P*
	B	Standard error	β		
Length of stay	−0.015	0.018	−0.062	−0.869	0.386
Delivery mode	−0.169	0.244	−0.049	−0.692	0.490
Days after birth	−0.021	0.015	−0.100	−1.405	0.162
Birth weight (kg)	0.027	0.134	0.014	0.202	0.840
Type of painful procedures	0.294	0.088	0.233	3.348	0.001
Sex	0.042	0.218	0.014	0.193	0.847
Apgar score at 1 min after birth	0.309	0.054	0.378	5.709	<0.001
GA	0.046	0.005	0.584	10.081	<0.001

Multiple linear regression analysis was then performed to investigate the independent influencing factors of neonatal pain responses. The results indicate that the Apgar score at 1 min after birth (*β* = 0.272, *p* < 0.001) and GA (*β* = 0.503, *p* < 0.001) were the only independent factors associated with neonatal pain responses (Table 4).

**Table 4:** Multiple linear regression analysis of neonatal pain response variables.

Predictors	Unstandardized coefficient		Standardized coefficient	*t*	*P*
	B	Standard error	β		
Constant quantity	−7.855	1.154		−6.806	<0.001
Days after birth	0.016	0.012	0.076	1.318	0.189
Type of painful procedures	0.142	0.073	0.112	1.955	0.052
Apgar score at 1 min after birth	0.222	0.047	0.272	4.683	<0.001
GA	0.039	0.005	0.503	8.346	<0.001

## 4 Discussion

The transduction of noxious stimuli into pain experiences is influenced by individual physiology, personal history, and social context (45). Despite growing discussions on the importance of contextual factors in neonatal pain responses, existing studies yield inconsistent results. In this work, we analyzed eight contextual factors among 198 neonates. Univariate and multivariate analyses revealed the considerable effect of the Apgar scores at 1 min, along with GA, on neonatal pain scores and responses.

In this study, neonates with greater GA were more responsive to pain, which is consistent with the findings of most research (18, 28, 46). Neonates with younger GA exhibited less pronounced pain responses, but this finding does not necessarily indicate that they experienced less pain. Clinical studies have reported that preterm neonates may fail to engage behavior-related networks due to immature cortical connectivity (47). Preterm infants exhibit poorer central nervous system development, weaker muscle strength (20), and a limited ability to express pain through behavior compared with term and post term infants (48). However, some studies have found that GA showed no statistically significant effect on neonatal pain scores. A study that used the Neonatal Facial Coding System as a measurement tool included 50 neonates with very low GA (<28 weeks) found no difference in pain the responses between neonates aged 23–25 weeks and those aged 26–28 weeks (22). Another study that used the Douleur Aiguě du Nouveau-né (DAN), which included 42 preterm and term infants, revealed no statistically significant effect of GA on neonatal pain scores (23). The heterogeneity of these findings may be attributed to differences in the study populations and the varying sensitivities of pain assessment tools to various types of pain responses. In extremely preterm infants (GA < 32 weeks), pain responses are primarily mediated by spinal reflex withdrawal (withdrawal reflex) rather than cortical processing (7). Given the underdevelopment of the cortical areas at this stage, specific brain activity following nociceptive stimuli is difficult to detect, and pain responses are commonly characterized by exaggerated withdrawal reflexes and extensive body movements (49). Therefore, pain assessment tools that primarily rely on facial expression indicators, such as the Neonatal Facial Coding System, or those that place greater emphasis on facial expressions within their scoring systems, such as the DAN scale, may struggle in comprehensively reflecting the diversity of pain responses in this population.

Consistent with other studies (21, 29, 50), we observed a positive correlation between the 1 min Apgar score and neonatal pain response. However, some researches have reported inconsistent results. Although a longitudinal study found no significant correlation between Apgar and NIPS scores in preterm infants, the significant increases in the NIPS scores with postmenstrual age of infants under multiple heel stick procedures indicated that younger or sicker infants displayed less robust pain response behaviors (51). Conversely, another study reported a negative correlation between the 1 min Apgar and the Bernese pain scale (BPSN) pain scores of preterm infants (12). These conflicting results may be due to differences in the pain responses of preterm infant. Morison et al. (29) reported that infants with low Apgar scores showed significantly increased body movement signals (such as limb extension assessed by Developmental Care and Assessment Program) after heel lance, whereas facial responses (measured by Neonatal Facial Coding System) were weaker. From a neurophysiological perspective, repeated pain stimuli can trigger the “wind-up” phenomenon in spinal dorsal horn cells, which amplifies reflexive motor responses. By contrast, low Apgar scores (which are possibly associated with perinatal hypoxia and central inhibition, such as cortical dysfunction) may reduce the transmission of pain signals to facial expressions, which leads to discrepancies in study findings.

The emphasis on different dimensions of pain response in scale items may affect the generalizability of research findings. The neurophysiological maturity of infants and pain expression pathways (spinal reflex vs. cortical integration) exhibit significant heterogeneity across different GAs, 1 min Apgar scores, and other clinical conditions (7, 49, 52). Therefore, the validity and accuracy of pain assessment tools showed a close linkage to their contextual adaptability. Currently, no single scale can be ideally applied to all types of pain or neonatal contexts. Particularly, under the influence of contextual factors, the expression and intensity of pain may vary. Although GA has been incorporated into various pain assessment tools (53–55), the extent of the effect of contextual factors on pain response remains to be further explored. In this study, we identified independent factors influencing neonatal pain responses through the analysis of extensive clinical data, and the results provide a basis for the future development of pain assessment tools and management strategies. Effective integration of these background factors into scale design and reasonable adjustment of the weight of various dimensions can facilitate accurate neonatal pain assessment.

### 4.1 Limitations

This study encountered some limitations. First, we performed data collection utilizing convenience sampling at a class-A tertiary hospital, where patients typically present with more intricate or severe diseases than those at smaller hospitals, hence constraining the generalizability of the sample. In addition, variations in clinical devices/equipment for painful procedures, differences in neonatal intensive care settings (e.g., noise, temperature, lighting, etc.), and the individual techniques of nurses performing procedures (e.g., heel sticks) possibly influenced the neonates' pain responses. In the future, multicenter studies may be undertaken at hospitals of differing grades/classes to enhance sample diversity and yield more generalizable results. Should the sample size be sufficiently high, subgroup analysis of infants with certain disorders, such as prematurity, will be crucial to distinguish and contextualize the pain response.

## 5 Conclusion

Neonatal pain responses are under the influence of contextual factors. The results of this study indicate that GA and the Apgar score at 1 min postdelivery are significantly associated factors of neonatal pain responses. The findings underscore the necessity of accounting for these factors in neonatal pain evaluation and emphasize the relevance of a holistic approach that amalgamates contextual data to enhance pain assessment in neonates. Further study is required to ascertain trustworthy parameters that can be integrated into neonatal pain evaluation and therapy protocols.

## Data Availability

The original contributions presented in the study are included in the article/Supplementary Material, further inquiries can be directed to the corresponding authors.

## References

[1] Çamur Z Erdoğan Ç. The effects of breastfeeding and breast milk taste or smell on mitigating painful procedures in newborns: systematic review and meta-analysis of randomized controlled trials. Breastfeed Med. (2022) 17(10):793–804. 10.1089/bfm.2022.0134 36126292

[2] Carbajal R Rousset A Danan C Coquery S Nolent P Ducrocq S Saizou C Epidemiology and treatment of painful procedures in neonates in intensive care units. JAMA. (2008) 300(1):60–70. 10.1001/jama.300.1.60 18594041

[3] McPherson C Grunau RE. Neonatal pain control and neurologic effects of anesthetics and sedatives in preterm infants. Clin Perinatol. (2014) 41(1):209–27. 10.1016/j.clp.2013.10.002 24524456 PMC3925313

[4] McPherson C Miller SP El-Dib M Massaro AN Inder TE. The influence of pain, agitation, and their management on the immature brain. Pediatr Res. (2020) 88(2):168–75. 10.1038/s41390-019-0744-6 31896130 PMC7223850

[5] Committee on Fetus and Newborn and Section on Anesthesiology and Pain Medicine. Prevention and management of procedural pain in the neonate: an update. Pediatrics. (2016) 137(2):e20154271. 10.1542/peds.2015-4271 26810788

[6] Maxwell LG Fraga MV Malavolta CP. Assessment of pain in the newborn: an update. Clin Perinatol. (2019) 46(4):693–707. 10.1016/j.clp.2019.08.005 31653303

[7] Relland LM Gehred A Maitre NL. Behavioral and physiological signs for pain assessment in preterm and term neonates during a nociception-specific response: a systematic review. Pediatr Neurol. (2019) 90:13–23. 10.1016/j.pediatrneurol.2018.10.001 30449602

[8] Walas W Latka-Grot J Szczapa T Maroszyńska I Rutkowska M Bartkowska-Śniatkowska A Usefulness of two types of pain monitors in newborns treated in NICU, in the opinion of experts: results of the survey. J Mother Child. (2022) 25(2):72–6. 10.34763/jmotherandchild.20212502.d-21-00018 34842396 PMC8976587

[9] Lee GY Stevens B. Neonatal and infant pain assessment. In: McGrath PJ Stevens BJ Walker SM Zempsky WT, editors. Oxford Textbook of Paediatric Pain. Oxford, United Kingdom: Oxford University Press (2014). p. 1.

[10] Schenk K Stoffel L Bürgin R Stevens B Bassler D Schulzke S Acute pain measured with the modified bernese pain scale for neonates is influenced by individual contextual factors. Eur J Pain. (2020) 24(6):1107–18. 10.1002/ejp.1555 32170786

[11] Cignacco E Schenk K Stevens B Stoffel L Bassler D Schulzke S Individual contextual factors in the validation of the bernese pain scale for neonates: protocol for a prospective observational study. BMC Pediatr. (2017) 17(1):171. 10.1186/s12887-017-0914-9 28724434 PMC5518104

[12] Sellam G Engberg S Denhaerynck K Craig KD Cignacco EL. Contextual factors associated with pain response of preterm infants to heel-stick procedures. Eur J Pain. (2013) 17(2):255–63. 10.1002/j.1532-2149.2012.00182.x 23203977

[13] Anand KJ. Consensus statement for the prevention and management of pain in the newborn. Arch Pediatr Adolesc Med. (2001) 155(2):173–80. 10.1001/archpedi.155.2.173 11177093

[14] Cong X McGrath JM Cusson RM Zhang D. Pain assessment and measurement in neonates: an updated review. Adv Neonatal Care. (2013) 13(6):379–95. 10.1097/ANC.0b013e3182a41452 24300956

[15] Maillard A Garnier E Saliba E Favrais G. Prematurity alters skin conductance and behavioural scoring after acute stress in term-equivalent age infants. J Acta Paediatr. (2019) 108(9):1609–15. 10.1111/apa.14777 30851198

[16] Yajing W Yang L. A study on the occurrence of procedural pain in neonates in the neonatal intensive care unit. J Nurs Manag. (2017) 17(11):797–800. 10.3969/j.issn.1671-315x.2017.11.010

[17] Sellam G Cignacco EL Craig KD Engberg S. Contextual factors influencing pain response to heelstick procedures in preterm infants: what do we know? A systematic review. Eur J Pain. (2011) 15(7):661.e1–15. 10.1016/j.ejpain.2011.01.002 21330173

[18] Grunau RE Oberlander TF Whitfield MF Fitzgerald C Lee SK. Demographic and therapeutic determinants of pain reactivity in very low birth weight neonates at 32 weeks’ postconceptional age. Pediatrics. (2001) 107(1):105–12. 10.1542/peds.107.1.105 11134442

[19] Gibbins S Stevens B. The influence of gestational age on the efficacy and short-term safety of sucrose for procedural pain relief. Adv Neonatal Care. (2003) 3(5):241–9. 10.1016/j.adnc.2003.08.004 14648521

[20] Gibbins S Stevens B McGrath PJ Yamada J Beyene J Breau L Comparison of pain responses in infants of different gestational ages. Neonatology. (2008) 93(1):10–8. 10.1159/000105520 17630493

[21] Johnston CC Stevens BJ. Experience in a neonatal intensive care unit affects pain response. Pediatrics. (1996) 98(5):925–30. 10.1542/peds.98.5.925 8909487

[22] Gibbins S Stevens B Beyene J Chan PC Bagg M Asztalos E. Pain behaviours in extremely low gestational age infants. Early Hum Dev. (2008) 84(7):451–8. 10.1016/j.earlhumdev.2007.12.007 18243593

[23] Yunli H Yongqing Y Dongming H Qiaozhen W Yuqi S Weiqiong W Clinical observation of factors affecting neonatal pain. Chin J Nurs. (2009) 44(08):709–11. 10.3761/j.issn.0254-1769.2009.08.013

[24] Valeri BO Gaspardo CM Martinez FE Linhares MB. Pain reactivity in preterm neonates: examining the sex differences. Eur J Pain. (2014) 18(10):1431–9. 10.1002/ejp.508 24733738

[25] Guinsburg R de Araújo Peres C Branco de Almeida MF de Cássia Xavier Balda R Cássia Berenguel R Tonelotto J Differences in pain expression between male and female newborn infants. Pain. (2000) 85(1–2):127–33. 10.1016/j.adnc.2003.08.004 10692611

[26] Petleshkova P Krasteva M Dragusheva S Bakova D Tornyova B Mihaylova A Factors affecting the severity of procedural pain in new-borns. J Spec Pediatr Nurs. (2018) 29:2049–52. 10.4066/biomedicalresearch.29-18-521

[27] Sharma R Sharma D. To study the effect of birth weight, gender and race on pain perception in neontes during vaccination. J Med Sci Clin Res. (2018) 6:897–901. 10.18535/jmscr/v6i2.138

[28] Schenk K Stoffel L Bürgin R Stevens B Bassler D Schulzke S Nelle M The influence of gestational age in the psychometric testing of the bernese pain scale for neonates. BMC Pediatr. (2019) 19(1):20. 10.1186/s12887-018-1380-8 30646872 PMC6334397

[29] Morison SJ Holsti L Grunau RE Whitfield MF Oberlander TF Chan HW Are there developmentally distinct motor indicators of pain in preterm infants? Early Hum Dev. (2003) 72(2):131–46. 10.1016/S0378-3782(03)00044-6 12782425

[30] Johnston CC Stevens B Yang F Horton L. Developmental changes in response to heelstick in preterm infants: a prospective cohort study. Dev Med Child Neurol. (1996) 38(5):438–45. 10.1111/j.1469-8749.1996.tb15101.x 8698151

[31] Bergqvist LL Katz-Salamon M Hertegård S Anand KJ Lagercrantz H. Mode of delivery modulates physiological and behavioral responses to neonatal pain. J Perinatol. (2009) 29(1):44–50. 10.1038/jp.2008.129 18769380

[32] Schuller C Känel N Müller O Kind AB Tinner EM Hösli I Stress and pain response of neonates after spontaneous birth and vacuum-assisted and cesarean delivery. Am J Obstet Gynecol. (2012) 207(5):416.e1–e6. 10.1016/j.ajog.2012.08.024 22959831

[33] Kassab M Hamadneh S Nuseir K ALmomani B Hamadneh J. Factors associated with infant pain severity undergoing immunization injections. J Pediatr Nurs. (2018) 42:e85–90. 10.1016/j.pedn.2018.04.002 29681431

[34] O'Neill MC Ahola Kohut S Pillai Riddell R Oster H. Age-related differences in the acute pain facial expression during infancy. Eur J Pain. (2019) 23(9):1596–607. 10.1002/ejp.1436 31162761

[35] Chen M Shi X Chen Y Cao Z Cheng R Xu Y A prospective study of pain experience in a neonatal intensive care unit of China. Clin J Pain. (2012) 28(8):700–4. 10.1097/AJP.0b013e3182400d54 22156826

[36] Kyololo OM Stevens B Gastaldo D Gisore P. Procedural pain in neonatal units in Kenya. Arch Dis Child Fetal Neonatal Ed. (2014) 99(6):F464–7. 10.1136/archdischild-2014-306003 24996597

[37] Zhu N Long B Zhan X Zhang L Wang Z Wang L Development of the neonatal pain response variable set: a mixed methods consensus process. Eur J Pediatr. (2024) 183(9):3719–26. 10.1007/s00431-024-05559-7 38850331 PMC11322254

[38] Lawrence J Alcock D McGrath P Kay J MacMurray SB Dulberg C. The development of a tool to assess neonatal pain. Neonatal Netw. (1993) 12(6):59–66. 10.1016/0885-3924(91)91127-u 8413140

[39] Motta Gde C Schardosim JM Cunha ML. Neonatal infant pain scale: cross-cultural adaptation and validation in Brazil. J Pain Symptom Manag. (2015) 50(3):394–401. 10.1016/j.jpainsymman.2015.03.019 26025270

[40] Xiaoli G Jing-Ru T Jing W Shu-Ming Pan Ying-Wei Wang. Bayesian estimation on diagnostic performance of face, legs, activity, cry, and consolability and neonatal infant pain scale for infant pain assessment in the absence of a gold standard. J Paediatric Anaesthesia. (2015) 25(8):834–9. 10.1111/pan.12664 25929312

[41] Suraseranivongse S Kaosaard R Intakong P Pornsiriprasert S Karnchana Y Kaopinpruck J A comparison of postoperative pain scales in neonates. Br J Anaesth. (2006) 97(4):540–4. 10.1093/bja/ael184 16885171

[42] Pediatrics A A o, Society C P. Prevention and management of pain in the neonate: an update. Pediatrics. (2006) 118(5):2231–41. 10.1542/peds.2006-2277 17079598

[43] Neonatology Branch of Chinese Medical Association; Editorial Board of Chinese Journal of Contemporary Pediatrics. Expert consensus on neonatal pain assessment and analgesic management (2020 edition). Chin J Contemp Pediatr. (2020) 22(09):923–30. 10.7499/j.issn.1008-8830.2006181 32933620 PMC7499443

[44] Bursac Z Gauss CH Williams DK Hosmer DW. Purposeful selection of variables in logistic regression. Source Code Biol Med. (2008) 3(1):17. 10.1186/1751-0473-3-17 19087314 PMC2633005

[45] Pillai Riddell R Fitzgerald M Slater R Stevens B Johnston C Campbell-Yeo M. Using only behaviours to assess infant pain: a painful compromise? Pain. (2016) 157(8):1579–80. 10.1097/j.pain.0000000000000598 27280549

[46] Gursul D Hartley C Slater R. Nociception and the neonatal brain. Semin Fetal Neonatal Med. (2019) 24(4):101016. 10.1016/j.siny.2019.05.008 31201139 PMC6728629

[47] Bucsea O Rupawala M Shiff I Wang X Meek J Fitzgerald M Clinical thresholds in pain-related facial activity linked to differences in cortical network activation in neonates. Pain. (2023) 164(5):1039–50. 10.1097/j.pain.0000000000002798 36633530 PMC10108588

[48] Valeri BO Linhares MBM. Pain in preterm infants: effects of sex, gestational age, and neonatal illness severity. Psychol Neurosci. (2012) 5(1):11–9. 10.3922/j.psns.2012.1.03

[49] Hartley C Moultrie F Gursul D Hoskin A Adams E Rogers R Changing balance of spinal cord excitability and nociceptive brain activity in early human development. Curr Biol. (2016) 26(15):1998–2002. 10.1016/j.cub.2016.05.054 27374336 PMC4985558

[50] Fangfei S Xiurong Y Yumei W Fang J Fang W. Analysis of pain scores and related factors for venipuncture in hospitalized preterm infants in the NICU. Qilu Nurs J. (2019) 25(18):83–7. 10.3969/j.issn.1006-7256.2019.18.030

[51] Williams AL Khattak AZ Garza CN Lasky RE. The behavioral pain response to heelstick in preterm neonates studied longitudinally: description, development, determinants, and components. Early Hum Dev. (2009) 85(6):369–74. 10.1016/j.earlhumdev.2009.01.001 19167172

[52] Grunau RE. Neonatal pain in very preterm infants: long-term effects on brain, neurodevelopment and pain reactivity. Rambam Maimonides Med J. (2013) 4(4):e0025. 10.5041/RMMJ.10132 24228168 PMC3820298

[53] Grunau RE Oberlander T Holsti L Whitfield MF. Bedside application of the neonatal facial coding system in pain assessment of premature neonates. Pain. (1998) 76(3):277–86. 10.1016/S0304-3959(98)00046-3 9718246

[54] Pölkki T Korhonen A Axelin A Saarela T Laukkala H. Development and preliminary validation of the neonatal infant acute pain assessment scale (NIAPAS). Int J Nurs Stud. (2014) 51(12):1585–94. 10.1016/j.ijnurstu.2014.04.001 24815773

[55] Stevens BJ Gibbins S Yamada J Dionne K Lee G Johnston C The premature infant pain profile-revised (PIPP-R): initial validation and feasibility. Clin J Pain. (2014) 30(3):238–43. 10.1097/AJP.0b013e3182906aed 24503979

